# Lactate quartile concentration and prognosis in severe sepsis and septic shock

**DOI:** 10.1186/cc13366

**Published:** 2014-03-17

**Authors:** M De La Torre-Prados, A Garcia-de la Torre, C Trujillano-Fernández, J Perez-Vacas, A Puerto-Morlan, E Camara-Sola, A Garcia-Alcantara, A Garcia-alcantara

**Affiliations:** 1 Hospital Virgen de la Victoria, Málaga, Spain; 2 Puerto Real University Hospital, Cadiz, Spain

## Introduction

The Surviving Sepsis Campaign (SSC) indicates that a lactate (LT) concentration greater than 4 mmol/l indicates early resuscitation bundles. However, several recent studies have suggested that LT values lower than 4 mmol/l may be a prognostic marker of adverse outcome. The aim of this study was to identify clinical and analytical prognostic parameters in severe sepsis (SS) or septic shock (ShS) according to quartiles of blood LT concentration.

## Methods

A cohort study was designed in a polyvalent ICU. We studied demographic, clinical and analytical parameters in 148 critically ill adults, within 24 hours from SS or ShS onset according to SSC criteria. We tested for differences in baseline characteristics by lactate interval using a Kruskal-Wallis test for continuous data or a chi-square test for categorical data and reported the median and interquartile ranges; SPSS version 15.0 (SPSS Inc., Chicago, IL, USA).

## Results

We analyzed 148 consecutive episodes of SS (16%) or ShS (84%). The median age was 64 (interquartile range, 48.7 to 71) years; male: 60%. The main sources of infection were respiratory tract 38% and intra-abdomen 45%; 70.7% had medical pathology. Mortality at 28 days was 22.7%. Quartiles of blood LT concentration were quartile 1 (Q1): 1.87 mmol/l or less, quartile 2 (Q2): 1.88 to 2.69 mmol/l, quartile 3 (Q3): 2.7 to 4.06 mmol/l, and quartile 4 (Q4): 4.07 mmol/l or greater (Table 1). The median LT concentrations of each quartile were 1.43 (Q1), 2.2 (Q2), 3.34 (Q3), and 5.1 (Q4) mmol/l (*P *< 0.001). The differences between these quartiles were that the patients in Q1 had significantly lower APACHE II scores (*P *= 0.04), SOFA score (*P *= 0.024), number of organ failures (NOF) (*P *< 0.001) and ICU mortality (*P *= 0.028), compared with patients in Q2, Q3 and Q4. Patients in Q1 had significantly higher cholesterol (*P *= 0.06) and lower procalcitonin (*P *= 0.05) at enrolment. At the extremes, patients in Q1 had decreased 28-day mortality (*P *= 0.023) and, patients in Q4 had increased 28-day mortality, compared with the other quartiles of patients (*P *= 0.009). Interestingly, patients in Q2 had significant increased mortality compared with patients in Q1 (*P *= 0.043), whereas the patients in Q2 had no significant difference in 28-day mortality compared with patients in Q3.

**Table 1:** Baseline characteristics in SS and ShS patients by quartiles of blood LT

value	Lactate <1.87 (*n *= 33)	Lactate 1.88 to 2.69 (*n *= 41)	Lactate 2.7 to 4.06 (*n *= 34)	Lactate>4.07 (*n *= 37)	*P*
Age (years)	57 (45 to 71)	64 (51.5 to 74.5)	65 (48 to 69)	60 (48.5 to 71)	NS
APACHE II	25 (18.5 to 30)	25 (19.5 to 27)	25 (21.5 to 29.5)	27 (22 to 33)	0.04
SOFA	9 (7 to 10.5)	9 (7 to 11)	9 (8 to 11)	11(8 to 13)	0.024
NOF	3 (3 to 4)	3 (3 to 4)	4 (3 to 5)	5 (3.5 to 5)	<0.001
LT (mmol/l)	1.43 (1.16 to 1.56)	2.2 (1.99 to 2.47)	3.34 (3 to 3.72)	5.1 (4.4 to 7.34)	<0.001
Procalcitonin (ng/ml)	2.81 (0.76 to 20.7)	11.5 (2.88 to 37.15)	13.47 (1.91 to 42.1)	21.6 (5.2 to 5.8)	0.05
Cholesterol (mg/dl)	127 (97.5 to 165)	130 (95.5 to 152.5)	100 (72 to 128)	91 (79 to 116.7)	0.06
28-day mortality (%)	10.8	21.2	24.4	35.1	0.029
ShS (%)	83.8	85.4	81.1	87.9	NS

## Conclusion

Adverse outcomes and several potential risk factors, including organ failure, are significantly associated with higher quartiles of LT concentrations. It may be useful to revise the cutoff value of lactate according to the SSC (4 mmol/l).

